# Do MRI Results Represent Functional Outcomes Following Arthroscopic Repair of an Isolated Meniscus Tear in Young Patients?—A Prospective Comparative Cohort Study

**DOI:** 10.3390/clinpract14020047

**Published:** 2024-04-01

**Authors:** Viktorija Brogaitė Martinkėnienė, Donatas Austys, Andrius Šaikus, Andrius Brazaitis, Giedrius Bernotavičius, Aleksas Makulavičius, Tomas Sveikata, Gilvydas Verkauskas

**Affiliations:** 1 Faculty of Medicine, Vilnius University, LT-03101 Vilnius, Lithuania; 2 Department of Children’s Orthopedics and Traumatology, Vilnius University Hospital Santaros Klinikos, LT-08406 Vilnius, Lithuania; 3 Department of Public Health, Institute of Health Sciences, Faculty of Medicine, Vilnius University, LT-03101 Vilnius, Lithuania; 4 Department of Radiology, Nuclear Medicine and Medical Physics, Faculty of Medicine, Vilnius University, LT-03101 Vilnius, Lithuania; 5 Centre for Radiology and Nuclear Medicine, Vilnius University Hospital Santaros Klinikos, LT-08661 Vilnius, Lithuania; 6 Clinic of Gastroenterology, Nefrourology and Surgery, Faculty of Medicine, Vilnius University, LT-03101 Vilnius, Lithuania; 7 Clinic of Rheumatology, Orthopaedics, Traumatology and Reconstructive Surgery, Faculty of Medicine Vilnius University, LT-03101 Vilnius, Lithuania

**Keywords:** knee, meniscal repair, functional outcomes, magnetic resonance image (MRI), young patients

## Abstract

Background: The use of postoperative MRI to assess the healing status of repaired menisci is a long-standing issue. This study evaluates and compares functional and MRI outcomes following an arthroscopic meniscus repair procedure with the aim of postoperative MRI diagnostic accuracy clarification in young patients. Methods: A total of 35 patients under 18 years old who underwent isolated meniscus repair were included. The Pedi-IKDC score, Lysholm score, and Tegner activity index (TAS) were compared between the groups formed according to the Stroller and Crues three-grade classification of postoperative MRI-based evaluations. Grade 3 MRI views were classified as unhealed, grade 2 as partially healed, and grade 1 as fully healed within the repaired meniscus, whereas grade 3 cases were considered unsuccessful due to MRI evaluation. Results: MRI assessment revealed 4 cases of grade 1 (11.4%), 14 cases of grade 2 (40.8%), and 17 cases of grade 3 (48.0%) lesions. Pedi-IKDC and TAS scores were significantly higher among MRI grade 2 patients than among MRI grade 3 patients (*p* < 0.05). Weak negative correlations between MRI grades and all functional scales were found (*p* < 0.05). ROC analysis showed that Pedi-IKDC and TAS scores could correctly classify 77% and 71% of MRI grade 3 patients, respectively. The optimal cut-off values to detect grade 3 patients were 88.74 for the Pedi-IKDC score and 4.5 for the TAS score. Conclusions: To conclude, established functional score cut-off values may help identify unhealed meniscus repair patients.

## 1. Introduction

The prevalence of meniscal injuries in adult patients is high [1]. It is less common in pediatric patients, although studies have shown that meniscal injuries are on the rise even in children population [2,3,4]. This might be attributed to improved diagnostic availability and increased engagement in high-demand sports activities, which is known as the most common cause of meniscus tear situations in skeletally immature patients [2,5]. Previously, the most prevalent method of treating this pathology was arthroscopic partial meniscectomy [1]. Over the recent decades, the importance of the meniscus as an intra-articular structure has been realized [5,6,7], and multiple studies have introduced long-term outcomes such as the accelerated development of degenerative osteochondral lesions after arthroscopic partial meniscectomy [8,9]. Therefore, the meniscus tear treatment has changed considerably in response [1,6]. To preserve as much meniscus tissue as possible, numerous repair methods were developed. Furthermore, it is crucial to provide appropriate treatment for meniscus tears in young individuals in order to prevent the eventual development of knee osteoarthritis following a meniscus injury [2]. Particularly in pediatric patients, meniscus repair procedures have been promising good and excellent outcomes due to better tissue healing potency [2,3].

The assessment of the meniscus healing process continues to be a matter of concern [7,8,9,10,11,12]. Clinical and functional outcomes are typically employed to assess the state of meniscus recovery [13]. In most cases, the functional and clinical condition of young individuals improves significantly after meniscus suturing [14,15,16]. However, if the patient continues to experience persistent symptoms following this type of treatment, further tests are conducted to assess the healing condition or retear of the meniscus [12]. In such cases, the magnetic resonance imaging (MRI) test is extensively utilized and readily available [12]. It has been widely accepted that clinical and imaging investigation prior to meniscus tear treatment is essential, and MRI has been the most popular and convenient diagnostic tool for detecting meniscus tears [3]. Preoperative MRI has been established to be highly reliable and accurate [3,17,18]. Inversely, the use of postoperative MRI for the assessment of the healing status of repaired menisci has long been debated by orthopedics and radiologists [9,10,11,19]. Several studies have demonstrated that MRI is not a reliable diagnostic tool for long-term follow-up evaluations of healing after meniscus suturing procedures [20,21]. Alternative diagnostic procedures such as magnetic resonance (MR) or computed tomography (CT) arthrography, as well as second-look arthroscopy, have been proposed as more effective than traditional MRI. However, their invasive nature and the associated risks of ionizing radiation make them unsuitable for routine usage, particularly in young patients [19,22,23,24]. Therefore, conventional MRI is still a test that is frequently referred to patients with functional restrictions or long-term clinical difficulties after a meniscus repair treatment because it is noninvasive and easily accessible. Since the MRI assessment is often used in order to determine the course of treatment, it is crucial to determine if the functional outcomes reflect the postoperative MRI imaging and to clarify the diagnostic accuracy of MRI after meniscal repair based on the patient’s functional outcomes. A lack of studies has been undertaken to investigate the connection between functional outcomes and postoperative MRI, and no study has been conducted on the subset of very young individuals who underwent meniscus repair. In order to distinguish clinically based failed cases and look for the connections to MRI results, we analyzed all cases using self-reported functional scores and MRI results.

Therefore, the aim of this prospective study is to evaluate and compare functional outcomes and MRI results with respect to MRI signal changes in pediatric patients after arthroscopic repair of an isolated meniscus tear.

## 2. Materials and Methods

A prospective study has been conducted under the agreement of the Vilnius Regional Bioethics Committee (Number 2021/51353825) and the Vilnius University Hospital Santaros Clinics Bioethics Committee. The study included patients with traumatic meniscus tears who met the study’s inclusion criteria and received arthroscopic meniscal surgery between May 2021 and December 2023 at the Children’s Orthopaedics and Traumatology Department of Vilnius University Hospital Santaros Clinics. Prior to enrollment, all patients’ parents or official carers gave informed consent. Twelve-year-old and older patients were required to provide additional consent. Age under 18 years old, a traumatic isolated full-thickness meniscus tear longer than 1 cm verified by preoperative MRI and arthroscopically, and no previous surgery on the injured knee were the inclusion criteria. Developmentally challenged patients and those not able to read or interpret Lithuanian were excluded from this study.

There were meniscus tears in two red–white and red–red zones, as well as a mix of them due to the meniscus’s bloodstream. Three anatomic components of a meniscus were determined: the anterior horn, the body, and the posterior horn. This classification was used to group all tears due to injury location in tears situated in the posterior horn, posterior horn-body, and all parts of the meniscus. Different meniscus injury patterns were involved.

### 2.1. Functional Evaluation

For functional knee evaluation, the Paediatric International Knee Documentation Committee (Pedi-IKDC) and Lysholm knee scores were applied, and the Tegner activity scale (TAS) was used to determine the patient’s degree of sports activity. All scores were fulfilled preoperatively and at the last follow-up, with a median duration of 13 months (11–15) in combination with a postoperative MRI examination.

### 2.2. MRI Evaluation Protocol

All participants of this study underwent postoperative MRI at the final follow-up, with a median of 13 months (11–15). The MRI was performed using 1.5-T MR equipment (SIGNA voyage system). Four main diagnostic sequences were analyzed, including sagittal proton density fast spin echo with fat saturation (Sag-PD FSE FS), sagittal T2-weighted fast spin echo (Sag-T2W FSE), coronal proton density fast spin echo with fat saturation (Cor- PD FSE FS), and coronal T2-weighted fast spin echo (Cor-T2W FSE), with the following parameters: the thickness of the slices was 3 mm, the repetition time ranged 2863 ms to 4389 ms, the field of view (FOV) was 180 mm, the gap between slices was 0.3 mm, the number of slices ranged from 28 to 31, and the total scan time was 15 min.

**Figure 1:**
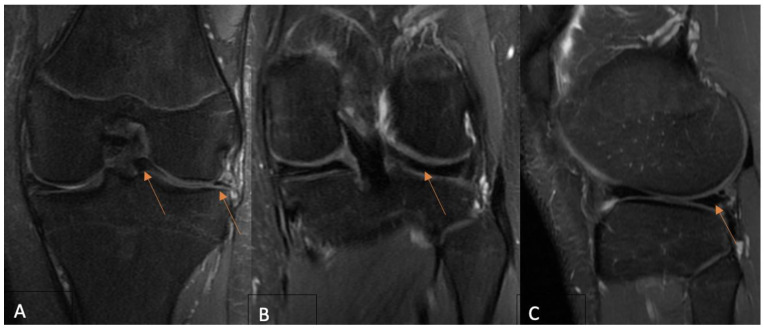
(**A**) A preoperative coronal PD-FSE, FS MR image shows (arrows) a bucket-handle-type lateral meniscus tear in a 16-year-old boy. Twelve-month postoperative coronal (**B**) and sagittal (**C**) PD-FSE, FS MR images reveal (arrows) a repaired lateral meniscus and no intrameniscal signal alterations—MRI grade 1 due to Crues and Stroller classification. MR—magnetic resonance; FSE—fast spin echo; FS—fat saturation.the figures (MRI views) indicate the meniscus structures in the knee joint and the tears (arrows) within the meniscus tissue.

**Figure 2:**
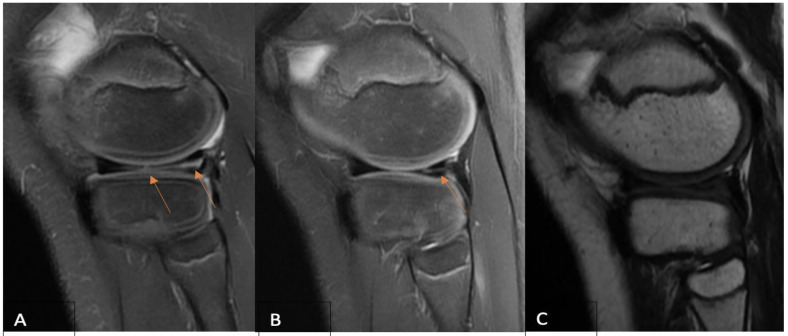
(**A**) A preoperative sagittal PD-FSE, FS MR imaging of an 11-year-old boy reveals (arrows) a complex (radial + longitudinal) type of lateral meniscus tear. Sagittal PD-FSE, FS (**B**), and T2-weighted FSE (**C**) MR images obtained 12 months after surgery, where sagittal PD-FSE, FS (**B**) indicates (arrow) a partially healed lateral meniscus with persisting intrameniscal signal changes that do not extend into the joint space, and sagittal T2-weighted FSE (**C**) shows no signal changes—MRI grade 2 due to Crues and Stroller grading. MR—magnetic resonance; FSE—fast spin echo; FS—fat saturation.

**Figure 3:**
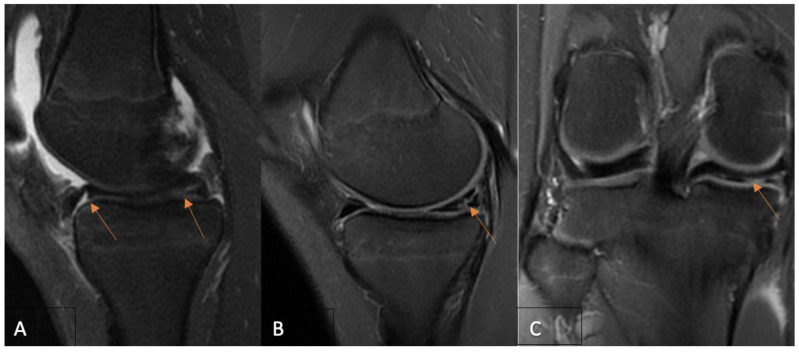
(**A**) A 14-year-old girl’s preoperative sagittal PD-FSE, FS MR imaging demonstrates (arrows) a medial meniscus tear of the bucket-handle type. Fourteen-month postoperative coronal (**B**) and sagittal (**C**) PD-FSE FS MR images establish (arrows) an unhealed medial meniscus with signal changes extending within the joint space—MRI grade 3 according to Crues and Stroller grading. MR—magnetic resonance; FSE—fast spin echo; FS—fat saturation.

Signal changes on postoperative MRI were graded using the Stoller and Crue three-stage classification [17,25]. Grade 0 was defined as a normal meniscus; the meniscus demonstrated low signal intensity in the images. Grade 1 was described as an intrameniscal signal with irregular margins that did not connect or communicate with an articular surface. Grade 2 was defined as a linear signal that did not abut or communicate with an articular surface. A linear or complex signal intensity that abutted or communicated with an articular surface was classified as grade 3. In summary, grade 3 was deemed unhealed, grade 2 partially healed, and grade 1 fully healed due to MRI assessment. A musculoskeletal imaging radiologist (A.B.) and an experienced orthopedic surgeon (A.Š.) performed the MRI evaluation independently and were blinded to functional evaluation. The intraclass correlation coefficient (ICC) was calculated for interobserver reliability. The overall consensus was reached for each case by both observers. Based on the MRI assessment, only cases with MRI grade 3 evaluation were considered unsuccessful. The grades of postoperative MRI for each of the three MRI groups are presented in Figure 1, Figure 2 and Figure 3.

### 2.3. Surgical Procedure

All participants in this study underwent arthroscopic meniscal repair surgery. A traditional two-portal approach was utilized. Three common suturing techniques were employed for meniscus repair, chosen based on the tear’s location and type, in order to provide optimal fixation and adaptability. The sutures were made either all-inside with internal anchors (Fast-Fix, Smith & Nephew) or inside–outside (Meniscus Needles, Arthrex) or outside–inside with a 2–0 number fiberwire suture and needles. No extra incisions were made if only the all-inside technique was used. Additional incisions between sutures on the skin were created in cases where either inside–outside or outside–inside techniques, or both, were performed. The sutures were orientated in various ways depending on the tear pattern, with the goal of aligning them vertically to enhance fixing strength. Table 1 shows the distribution of suturing methods and the number of sutures.

### 2.4. Statistics

Statistical analysis was performed with SPSS 24.0 IBM. The normality of the variables’ distribution was tested using the Shapiro–Wilk test. Because of the absence of normally distributed variables, nonparametric tests were used. Spearman correlation was used to analyze the relationship between MRI results and functional scales. The Mann–Whitney test was employed to compare the functional scores, patients’ age, and length of follow-up time according to the grade determined by the MRI. The chi-square test was used to assess the association between the grades determined with MRI and patients’ gender, meniscus side, meniscus injury location, and meniscus injury pattern. The Wilcoxon rank test was used to compare functional outcomes before the treatment and at the last follow-up. A significance level of 0.05 was used to reject the null hypothesis. Measures of central tendency were presented as follows: median (first quartile–third quartile). The Youden indexes from the receiver operator characteristic (ROC) curve analysis were conducted to determine the cut-off values of functional scales for classifying the MRI grade 3 patients. The area under the curve (AUC) and its 95% confidence intervals (CI) for the ROC curve were calculated to assess the diagnostic power of each functional scale. The ICC degree of agreement was categorized as follows: >0.80, almost excellent reproducibility; 0.61 to 0.80, good reproducibility; 0.41 to 0.60, moderate reproducibility; and 0.40, poor reproducibility.

## 3. Results

This study included 35 patients (35 menisci). More than half of them—60.0% (n = 21)—were boys, and 40.0% (n = 14) were girls. The median age was 16 (15–17) years. The median of injury-to-repair time was 8 (4–24) weeks. The medial meniscus was injured in 24 cases and lateral in 11 cases. The distribution of the injury pattern of meniscal injuries was as follows: bucket handles 16 (45.7%), vertical-longitudinal 9 (25.7%), horizontal cleavage 2 (5.7%), and 8 (22.9%) were complex-type tears. The posterior horn was involved in 17 cases, the posterior body in 15 cases, and in 3 cases, the hole length of the meniscus was involved. Table 1 provides the descriptive data that were collected.

Pedi-IKDC and Lysholm functional scores increased significantly after the procedure from 42.38 to 91.84 (*p* < 0.001) and 58.05 to 94 (*p* < 0.001), respectively. The TAS median score was 4 (3–7) preoperatively and 4 (3–6) at the most recent follow-up (*p* = 0.002). According to the TAS score, 66% of the patients have returned to preinjury activity level. Postoperative MRI assessment revealed 4 cases of grade 1 (11.4%), 14 cases of grade 2 (40.8%), and 17 cases of grade 3 (48.0%) lesions. Interobserver reliability based on the intraclass correlation coefficient (ICC) was good (ICC = 0.74, 0.46–0.86).

Pedi-IKDC and TAS scores were significantly higher among MRI grade 2 patients than among MRI grade 3 patients (respectively, *p* = 0.018, *p* = 0.021). Additionally, the Pedi-IKDC score was significantly higher among MRI grade 1 patients compared with MRI grade 3 patients (*p* = 0.02). Table 2 provides the entire statistical differences between the MRI groups due to functional scores. Table 3 indicates the comparison of MRI groups based on patients’ age, gender, meniscus side, time from injury to repair time, meniscus injury location, meniscus injury pattern, suture technique, suture number, and length of follow-up time. Spearman correlation coefficients were calculated between all functional scores and the MRI grades. The correlations were found to be significant (*p* < 0.05). The negative weak correlations between MRI grades and all functional scales were found: for the Pedi-IKDC scale, R = −0.498; Lysholm scale, R = −0.32; and TAS scale R = −0.323, *p* < 0.05.

**Figure 4:**
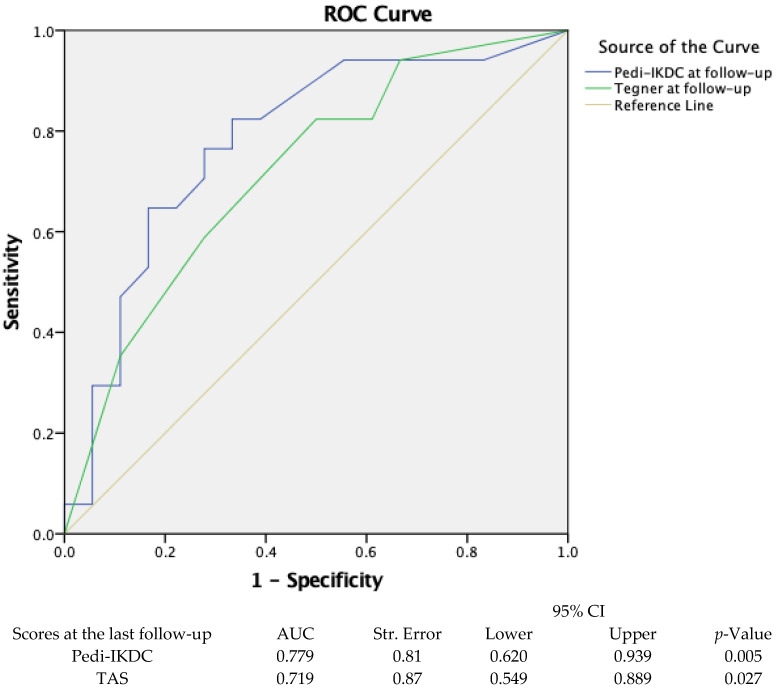
The application of ROC curve analysis to classify individuals with MRI grade 3 based on the Pedi-IKDC and TAS scores. Pedi-IKDC—Paediatric International Knee Documentation Committee; AUC—area under curve; TAS—Tegner activity score; ROC—receive operating characteristic.

The ROC curve analysis was performed to classify patients with MRI grade 3 who were estimated to have unsuccessful outcomes after meniscus repair surgery based on MRI scans. The statistically significant classification of MRI grade 3 patients was achieved only on Pedi-IKDC and TAS scores (respectively, *p* = 0.005 and *p* = 0.027). Respectively, the ROC analysis and the area under the ROC curve revealed that Pedi-IKDC and TAS scores could correctly classify 77% and 71% of the MRI grade 3 patient group defined by MRI staging. The areas under the curve for both scores were >0.7, and thus, they were considered acceptable. The optimal cut-off values for the classification of MRI grade 3 patients based on MRI results were 88.74 and 4.5 for the Pedi-IKDC and TAS scores, respectively. The statistical data and graphical representation of ROC analyses are presented in Figure 4.

## 4. Discussion

The current study was conducted to reveal if postoperative MRI findings and functional outcomes correlate, with the goal of analyzing conventional MRI signal changes based on functional scores after a repaired meniscus in young patients. In our study, like in many others, the preoperative functional scores altered significantly following the meniscus repair operation. However, the primary focus of this study was on comparing these functional outcomes with MRI data, rather than on meniscus repair’s overall success rates, which are widely disparate in the literature [13]. As a result, the evaluation of MRI views was the first critical step in our research. The MRI staging classification chosen in our study is the most widely used and is based on histological evidence of meniscal injuries [17,25]. The MRI findings in our study indicated that almost 50% of the meniscus remained unhealed, classified as grade 3. Other studies have demonstrated similar MRI conclusions following a meniscus repair procedure [11,18,26]. The primary objective of this study was to analyze potentially significant differences between all three MRI grades of patients regarding all functional scores. The comparison of the functional scores with respect to the MRI grades demonstrated that individuals with MRI grade 2 had significantly higher Pedi-IKDC and TAS scores compared with those with MRI grade 3. This implies a link between functional outcomes and MRI results. However, the weak correlations revealed in our study do not provide evidence of a strong concordance between MRI results and functional outcomes. As a consequence, our study’s focus turned to patients with MRI grade 3 findings who were judged as unhealed within the repaired meniscus and deemed as failed cases based on MRI assessment in this study. The ROC curve analysis demonstrated that the classification of MRI grade 3 patients based on Pedi-IKDC and TAS scores was found to be statistically significant. This investigation specifically focused on distinguishing between grade 2 and grade 3 patients. The area under the curve showed a greater level of agreement of MRI grade 3 patients with the Pedi-IKDC score compared with the TAG score, indicating a slight overall classification power. In our opinion, the most practical part of the ROC analysis was the determination of the optimal cut-off values for the scores, which were found to be useful in the classification of the MRI grade 3 patients. According to the findings, individuals who self-report a Pedi-IKDC score of up to 88.7 and a TAS score of up to 4.5 could be detected as MRI grade 3 patients on postoperative MRI images and suspected of having unhealed tissue within the treated meniscus.

The importance of this topic is primarily related to the meniscus repair procedure, which is currently performed on a regular basis in young, active patients and has highly promising overall outcomes [13]. Consequentially, the postoperative MRI findings are important since young patients used to experience problems after meniscus repair surgery. Shiehel et al. found that the revision rate was primarily related to extremely young age (open physis) and bucket-handle tear pattern, which were prevalent in this study [27]. The same author also discovered that the majority of failures in that age group of patients are the consequence of an acute reinjury within 1 year [28]. These circumstances necessitate a convenient and relevant investigation of the patient and the interpretation of the findings.

To the date, many authors have shown that the postoperative conventional MRI is less reliable than the preoperative MRI in diagnosing meniscus tears [9,17,20,21]. It might be due to the scar tissue formation process, and the signal changes might last for many years, even permanently [23]. As suggested by Miao et al., the diagnostic accuracy of the postoperative MRI could be enhanced through an extended follow-up period [9]. Therefore, the comparatively short mean follow-up period in our study may have an impact on the MRI results. An additional aspect notable of consideration pertains to the categorization of grade 3 patients in accordance with the original Stroller and Crues structure. Both the previous author and Hoffelner et al. proposed in their respective articles an approach of assessing the signal’s intensity and pairing it with the articular fluid signal in MRI grade 3 patients. As a result, if the signal intensity is reduced due to fluid intensity, the meniscus is likely to be healed [9,18].

Additionally, it is widely recognized that postoperative MRI is less efficient than second-look arthroscopy or MR/CT arthrography in assessing the healing status of a repaired meniscus [19,22,23]. The second-look arthroscopy, if there are any postoperative issues, is the most accurate test, which remains the “gold standard” [19]. There are many studies about the comparison of the MRI findings and the second-look arthroscopy results [9,10,11,19,29,30]. In most cases, the same conclusion is provided: the results of the postoperative MRI and the second-look arthroscopy differ. The overall second-look arthroscopy results are superior to postoperative MRI findings [9,19,29]. However, these methods of testing the knee are less common due to well-known disadvantages, like as invasiveness, time, radiation, and other factors. It could hardly be performed on a regular basis, especially in younger individuals [10,15]. Based on the findings of Yamasaki et al. [11], MRI mapping can be an effective method for meniscal healing evaluation. Although it is still challenging to conduct in everyday practice, this particular sort of MRI gives the benefit of not being invasive. Despite the fact that there are more effective techniques for evaluating the healing process of the meniscus after suturing, the standard MRI is still commonly performed as the initial diagnostic test if symptoms such as joint swelling or pain remain [10,18,20]. Therefore, standard MRI analyses continue to catch everyone’s interest. To accomplish this purpose, Schwach et al. suggested evaluating signal intensity, morphological changes, and tear diastasis alterations rather than classifying MRI results using the Stroller and Crues classification [12]. A negative correlation was discovered between the tear diastasis and the functional scores, demonstrating that a smaller gap after surgery indicated the healing of the meniscus. Our study also revealed negative correlations between the functional scores and MRI grades, indicating a correlation between higher functional scores and greater meniscus healing as measured by MRI grades. Additionally, a number of authors examine the accuracy of MRI by investigating clinical outcomes [29,30,31]. Fauno et al. assessed conventional MRI accuracy by employing clinical criteria and reported that MRI contributes to enhancing the diagnostic accuracy of an unhealed meniscal repair when fewer than three clinical symptoms persist [30]. Only a few studies compared MRI data and functional scores [12,18], perhaps due to the fact that it could be very different in clinical practice. However, this approach of analyzing the postoperative MRI remains very interesting in its practical application.

Several limitations occurred in this study. A strict patient selection process was followed for the initial group, leading to the relatively small sample size in this study. However, the study only included young individuals who have experienced a traumatic meniscus tear and did not have any concomitant knee lesions at the same time. Nevertheless, the limited number of cases could potentially affect the statistical outcomes. Nonetheless, it seems to be similar to those mentioned in the literature [15,18,32,33,34]. The meniscus tear pattern subgroup was left out from the analysis due to the small sample size. Another disadvantage is the 1.5T MRI system, which is frequently replaced by more advanced 3T equipment these days. However, Hoffelnner et al. have found that the evaluation of the meniscus healing process after the meniscus repair procedure does not differ between the systems [18]. An additional underlying weakness of this study is the absence of verification using second-look arthroscopy or MR/CT arthrography, which are recognized as superior techniques for assessing the success rate of meniscus healing.

## 5. Conclusions

MRI evaluations of the healing process of meniscus in patients with previously repaired menisci correlate with the functional scores. Patients with MRI grade 2 demonstrated significantly higher functional outcomes than those with MRI grade 3, as measured by Pedi-IKDC and TAS scores. Similarly, patients identified as MRI grade 1 demonstrated significantly superior functional outcomes than those classified as MRI grade 3 according to the Pedi-IKDC scale. The application of established functional score cut-off values could assist in the identification of MRI grade 3 patients and suspected unhealed meniscus.

## Figures and Tables

**Table 1:** The characteristics of every participant of this study.

Case	Sex	Age/y	Side	Sutures	Meniscal Injury Location	Tear Pattern	Technique	MRI Grade	Time to Operation/w
1	b	16	lateral	3	P-M	complex	all inside	3	20
2	b	15	lateral	4	P-M	bucket handle	all inside	2	12
3	b	15	medial	6	A-P	bucket handle	hybrid	3	4
4	g	12	medial	4	P-M	bucket handle	hybrid	3	4
5	b	15	lateral	2	P	bucket handle	all inside	3	8
6	g	17	medial	2	P	bucket handle	all inside	3	33
7	g	16	lateral	4	P-M	bucket handle	hybrid	2	3
8	b	15	medial	1	P	longitudinal	all inside	2	3
9	b	17	medial	5	P	bucket handle	hybrid	1	4
10	g	16	medial	1	P	longitudinal	all inside	2	4
11	g	15	medial	6	P-M	bucket handle	hybrid	2	3
12	g	17	medial	4	A-P	bucket handle	hybrid	2	1
13	b	16	medial	2	P	vertical–longitudinal	all inside	3	8
14	b	17	medial	3	P	bucket handle	hybrid	2	1
15	b	15	lateral	3	P	bucket handle	hybrid	2	24
16	g	16	medial	3	P	horizontal	all inside	3	20
17	b	17	medial	2	P	longitudinal	all inside	2	77
18	g	17	medial	3	P-M	complex	all inside	2	6
19	g	17	medial	2	P	longitudinal	all inside	3	25
20	b	11	lateral	2	P-M	complex	all inside	2	4
21	g	15	lateral	5	P-M	bucket handle	hybrid	3	12
22	g	17	medial	3	P-M	complex	hybrid	3	12
23	b	17	medial	3	P-M	complex	all inside	2	2
24	b	16	lateral	2	P-M	horizontal	all inside	1	8
25	b	14	medial	3	P	bucket handle	all inside	3	16
26	g	14	medial	1	P	vertical–longitudinal	all inside	3	52
27	g	17	medial	2	P	longitudinal	all inside	2	12
28	b	17	medial	5	A-P	bucket handle	hybrid	3	2
29	b	17	lateral	1	P	complex	all inside	3	8
30	b	15	lateral	2	P	longitudinal	all inside	2	24
31	b	14	medial	2	P-M	complex	all inside	3	36
32	b	16	medial	3	P-M	bucket handle	all inside	1	4
33	b	15	medial	2	P-M	complex	hybrid	3	32
34	b	17	medial	2	P	longitudinal	all inside	1	24
35	g	14	lateral	4	P-M	bucket handle	hybrid	3	2

Abbreviations: b—boy; g—girl; MRI—magnetic resonance imaging; y—years; w—weeks; P—posterior horn of the meniscus; P-M—posterior horn and middle part of the meniscus; A-P—all parts of the meniscus.

**Table 2:** The comparison of patient MRI groups according to different functional scores.

		Pedi-IKDC Mdn (Q1–Q3) 92.39 (84.78–97.82)	Lysholm Mdn (Q1–Q3) 94 (85–100)	Tegner Mdn (Q1–Q3) 4 (3–7)
**MRI grade 1/MRI grade 2**	*p*	0.88	0.75	0.83
**MRI grade 2/MRI grade 3**	*p*	0.018	0.27	0.021
**MRI grade 1/MRI grade 3**	*p*	0.02	0.061	0.35

Abbreviations: MRI—magnetic resonance imaging; Pedi-IKDC—Paediatric International Knee Documentation Committee score; Lysholm—knee scoring, Tegner—Tegner activity index; *p*—*p*-value; Mdn—median; Q1—first quartile, Q3—third quartile.

**Table 3:** The comparison of MRI groups of patients based on their characteristics.

		MRI Grades Grade 1 N = 4 (11.4%) Grade 2 N = 14 (40.8%) Grade 3 N = 17 (48.0%)		
**Gender**				
boys N = 21 (60.0%)/ girls N = 14 (40.0%)	*p*	ns		
**Meniscal side**				
medial N = 24 (68.6%)/ lateral N = 11 (31.4%)	*p*	ns		
**Meniscus injury location**				
back and middle N = 15 (42.9%)/ back N = 17 (48.6%)/ all long N = 3 (8.6%)	*p*	ns		
**Meniscus tear pattern**				
longitudinal N = 9 (25.7%)/ bucket handle N = 16 (42.7%)/ horizontal N = 2 (5.7%)/ complex N = 6 (22.6%)	*p*	ns		
**Suture technique**				
all-inside N = 22 (62.9%)/ hybrid N = 13 (37.1%)	*p*	ns		
		**MRI Grade 1/MRI Grade 2**	**MRI Grade 2/MRI Grade 3**	**MRI Grade 1/MRI Grade 3**
**Age**	*P*	0.36	0.32	0.13
Years/Mds (Q1–Q3) 16 (15–17)				
**Follow-up**	*p*	0.83	0.21	0.44
Months/Mds (Q1–Q3) 13 (11–15)				
**Injury to repair time**	*p*	0.45	0.07	0.34
Weeks/Mds (Q1–Q3) 8 (4–24)				
**Suture number** Mds (Q1–Q3) 3 (2–4)	*p*	0.91	0.95	0.92

Abbreviations: MRI—magnetic resonance imaging; *p*—*p*-value; ns—not significant; Mdn—median; Q1—first quartile; Q3—third quartile; N—number of cases.

## Data Availability

The data presented in this study are available on request from the corresponding author (viktorija.brogaite@santa.lt) due to study protocol regulations.

## References

[1] Bhan K. (2022). Meniscal Tears: Current Understanding, Diagnosis, and Management. Cureus.

[2] Asokan A., Ayub A., Ramachandran M. (2023). Pediatric meniscal injuries: Current concepts. J. Child. Orthop..

[3] Cabral J., Sinikumpu J. (2023). Clinical considerations of anatomy and magnetic resonance imaging in pediatric meniscus tear, with imaging-based treatment options. J. Child. Orthop..

[4] Popper H.R., Fliegel B.E., Elliott D.M., Su A.W. (2023). Surgical Management of Traumatic Meniscus Injuries. Pathophysiology.

[5] Vinagre G., Cruz F., Alkhelaifi K., D’Hooghe P. (2022). Isolated meniscus injuries in skeletally immature children and adolescents: State of the art. J. ISAKOS.

[6] Kopf S., Beaufils P., Hirschmann M.T., Rotigliano N., Ollivier M., Pereira H., Verdonk R., Darabos N., Ntagiopoulos P., Dejour D. (2020). Management of traumatic meniscus tears: The 2019 ESSKA meniscus consensus. Knee Surg. Sports Traumatol. Arthrosc..

[7] Fedje-Johnston W., Johnson C.P., Tóth F., Carlson C.S., Ellingson A.M., Albersheim M., Lewis J., Bechtold J., Ellermann J., Rendahl A. (2021). A pilot study to assess the healing of meniscal tears in young adult goats. Sci. Rep..

[8] Li C.A., Kim M.K., Kim I.H., Lee J.H., Jang K.Y., Lee S.Y. (2013). Correlation of Histological Examination of Meniscus with MR Images: Focused on High Signal Intensity of the Meniscus Not Caused by Definite Meniscal Tear and Impact on MR Diagnosis of Tears. Korean J. Radiol..

[9] Miao Y., Yu J.K., Zheng Z.Z., Yu C.L., Ao Y.F., Gong X., Wang Y.J., Jiang D. (2009). MRI signal changes in completely healed meniscus confirmed by second-look arthroscopy after meniscal repair with bioabsorbable arrows. Knee Surg. Sports Traumatol. Arthrosc..

[10] Song B., Tan W., Xu Y., Yu T., Li W., Chen Z., Yang R., Hou J., Zhou Y. (2019). 3D-MRI combined with signal-to-noise ratio measurement can improve the diagnostic accuracy and sensitivity in evaluating meniscal healing status after meniscal repair. Knee Surg. Sports Traumatol. Arthrosc..

[11] Yamasaki S., Hashimoto Y., Nishida Y., Teraoka T., Terai S., Takigami J., Nakamura H. (2020). Assessment of Meniscal Healing Status by Magnetic Resonance Imaging T2 Mapping After Meniscal Repair. Am. J. Sports Med..

[12] Schwach M., Grange S., Klasan A., Putnis S., Philippot R., Neri T. (2023). MRI Criteria for Healing at 1 Year After Repair of a Traumatic Meniscal Tear. Am. J. Sports Med..

[13] Yang B.W., Liotta E.S., Paschos N. (2019). Outcomes of Meniscus Repair in Children and Adolescents. Curr. Rev. Musculoskelet. Med..

[14] Milliron E.M., Magnussen R.A., ACavendish P., PQuinn J., DiBartola A.C., Flanigan D.C. (2021). Repair of Radial Meniscus Tears Results in Improved Patient-Reported Outcome Scores: A Systematic Review. Arthrosc. Sports Med. Rehabil..

[15] Muench L.N., Achtnich A., Krivec L., Diermeier T., Woertler K., Braun S., Imhoff A.B., Willinger L. (2022). Clinical outcome and healing rate after meniscal bucket handle tear repair. BMC Musculoskelet. Disord..

[16] Willinger L., Herbst E., Diermeier T., Forkel P., Woertler K., Imhoff A.B., Achtnich A. (2019). High short-term return to sports rate despite an ongoing healing process after acute meniscus repair in young athletes. Knee Surg. Sports Traumatol. Arthrosc..

[17] Lefevre N., Naouri J.F., Herman S., Gerometta A., Klouche S., Bohu Y. (2016). A Current Review of the Meniscus Imaging: Proposition of a Useful Tool for Its Radiologic Analysis. Radiol. Res. Pract..

[18] Hoffelner T., Resch H., Forstner R., Michael M., Minnich B., Tauber M. (2011). Arthroscopic all-inside meniscal repair—Does the meniscus heal?: A clinical and radiological follow-up examination to verify meniscal healing using a 3-T MRI. Skelet. Radiol..

[19] Morgan C.D., Wojtys E.M., Casscells C.D., Casscells S.W. (1991). Arthroscopic meniscal repair evaluated by second-look arthroscopy. Am. J. Sports Med..

[20] Muellner T., Egkher A., Nikolic A., Funovics M., Metz V. (1999). Open Meniscal Repair: Clinical and Magnetic Resonance Imaging Findings After Twelve Years. Am. J. Sports Med..

[21] Pujol N., Tardy N., Boisrenoult P., Beaufils P. (2013). Magnetic Resonance Imaging is not suitable for interpretation of meniscal status ten years after arthroscopic repair. Int. Orthop..

[22] Tapasvi S., Shekhar A., Chandorkar A., Patil A., Patil S. (2021). Indirect Magnetic Resonance Arthrography May Help Avoid Second Look Arthroscopy for Assessment of Healing After Bucket Handle Medial Meniscus Repairs: A Prospective Clinico-Radiological Observational Study. Indian. J. Orthop..

[23] Vance K., Meredick R., Schweitzer M.E., Lubowitz J.H. (2009). Magnetic Resonance Imaging of the Postoperative Meniscus. Arthrosc. J. Arthrosc. Relat. Surg..

[24] Baker J.C., Friedman M.V., Rubin D.A. (2018). Imaging the Postoperative Knee Meniscus: An Evidence-Based Review. Am. J. Roentgenol..

[25] Stoller D.W., Martin C., Crues J.V., Kaplan L., Mink J.H. (1987). Meniscal tears: Pathologic correlation with MR imaging. Radiology.

[26] Zhu S., Li X., Lu Z., Koh J.L., Wang C., Wang P., Shao X., Wang J. (2023). Arthroscopic repair of degenerative medial meniscus tears in patients aged over 45 years resulted in favorable clinical outcomes and low clinical failure rates at a minimum 2-year follow-up. Knee Surg. Sports Traumatol. Arthrosc..

[27] Shieh A., Bastrom T., Roocroft J., Edmonds E.W., Pennock A.T. (2013). Meniscus Tear Patterns in Relation to Skeletal maturity: Children Versus Adolescents. Am. J. Sports Med..

[28] Shieh A.K., Edmonds E.W., Pennock A.T. (2016). Revision Meniscal Surgery in Children and Adolescents: Risk Factors and Mechanisms for Failure and Subsequent Management. Am. J. Sports Med..

[29] Miao Y., Yu J.K., Ao Y.F., Zheng Z.Z., Gong X., Leung K.K.M. (2011). Diagnostic Values of 3 Methods for Evaluating Meniscal Healing Status After Meniscal Repair: Comparison Among Second-Look Arthroscopy, Clinical Assessment, and Magnetic Resonance Imaging. Am. J. Sports Med..

[30] Faunø E., Sørensen O.G., Nielsen T.G., Lind M., Tvedesøe C. (2020). Magnetic resonance imaging can increase the diagnostic accuracy in symptomatic meniscal repair patients. Knee Surg. Sports Traumatol. Arthrosc..

[31] Steenbrugge F., Verstraete K., Verdonk R. (2004). Magnetic reasonance imaging of the surgically repaired meniscus: A 13-year follow-up study of 13 knees. Acta Orthop. Scand..

[32] Schmitt A., Batisse F., Bonnard C. (2016). Results with all-inside meniscal suture in pediatrics. Orthop. Traumatol. Surg. Res..

[33] Lucas G., Accadbled F., Violas P., Sales de Gauzy J., Knörr J. (2015). Isolated meniscal injuries in paediatric patients: Outcomes after arthroscopic repair. Orthop. Traumatol. Surg. Res..

[34] Isono M., Koga H., Nakagawa Y., Nakamura T., Sekiya I., Katagiri H. (2023). Inferior Healing Rate in Isolated Meniscal Repair than that in Meniscal Repair with Concomitant ACL Reconstruction Evaluated with MRI. Malays Orthop. J..

